# "The Hospital" Nursing Mirror

**Published:** 1900-10-20

**Authors:** 


### "The Hospital" Nursing Mirror.

BEING THE NURSING SECTION OF "THE HOSPITAL."

[Contributions for this Section of "THE HOSPITAL" should be addressed to the Editor, "THE HOSPITAL" NURSING MIRROR, 28 & 29 Southampton Street, Strand, London, W.C.]

### Notes on News from the Nursing World.

### OUR CHRISTMAS DISTRIBUTION.

WE have already received one parcel of clothing for our Christmas distribution, but we are unable to acknowledge it in the usual manner, as the only indication of the sender is that it was despatched from Marloes Road, South Kensington. Of course we are pleased to receive gifts anonymously, but we again invite our friends who wish to know that their parcels have safely reached us, to put their name and address inside.

### THE HOSPITAL SHIP "AVOCA."

ALTHOUGH there were two deaths on board the "Avoca" during its recently completed passage home, the ship had an excellent passage. Major Rose, R.A.M.C., was in medical charge, and five civilian doctors were on duty; Sister Makepeace, A.N.S., was superintendent, as she has been since the ship was fitted as a hospital ship; and with six A.N.S.R. Sisters made up the female nursing staff; while a staff sergeant, one corporal, and two privates R.A.M.C., two sergeants of Imperial Hospital Corps, two sergeants and thirty men of St. John Ambulance Brigade composed the male nursing staff. At Southampton a special train was brought to the shed alongside of the ship, and the patients were taken to Netley with every comfort and convenience for the lying-down cases. Those who were able went to their own depots, as most had greatly improved in health during the voyage home. All patients on board expressed their appreciation and gratitude for the kindness and courtesy shown them during the voyage by thanking the commanding officer, Captain Fausset, and officers of the ship at a concert which took place before the vessel got into the Bay. One of the most interesting cases on board was that of a 5th Dragoon Guardsman, who had been wounded—shot through the chest. He found himself taken prisoner by the Boers, who treated him most shamefully, breaking both his arms by blows from a rifle, so that he should be rendered the more helpless.

### THE HOURS OF DUTY ON THE "AVOCA."

ONE of the nursing sisters on board the "Avoca" speaks in warm terms of the excellent food, "good in quality and quantity," which was served to the men. She says that she never heard a grumble, and that many remarked to her that they had no possible cause for complaint. "Our hours," she proceeds, "were fairly long—on duty at 7.20 a.m. until 8.45; breakfast at 9, wards again 9.45, to be all ready for the doctor at 10. Tea and biscuits were sent to the pantry at 11, and we were off duty at 12.30 (if possible) for lunch at 1. At 2 o'clock we went down to give medicine, &c., and went off at 2.30, except the sister on duty, who went round at 3 and 4, or oftener if needed. We had tea at 4, and went on duty at 5.30 until 6.45. Dinner was at 7, and we were on duty from 7.45 to 8.30. We had two officers on board who had been through the Ladysmith siege, and who were still far from strong; of course they had had enteric. Each man was served with an entire outfit of warm underclothing before landing, with cholera belts to any who needed them. I shall be very glad to hear that I am going back in the same ship. Everyone was so kind, and tried to make the voyage pass pleasantly."

### NURSES ON THE TREATMENT OF THE SOLDIERS.

A SISTER who has just returned from South Africa writes us: "In Natal, where I was nursing, all that was possible was done for the comfort of the soldiers. We even had the luxury of baths. As the water supply was good, baths and washhouses were fitted up where the convalescent patients could indulge freely, a boon which, I can assure you, was appreciated. The patients also had as much clean linen as was necessary—which was the most missed by my patients in the field hospital I had been in previously." Sister Makins, Royal Red Cross, has written to one of the daily papers to testify to the continuous kindness shown to the wounded by the Colonial ladies while she was nursing for several months in Cape and Orange River Colonies. "In No. 1 Hospital, Wynberg," she says, "we had an almost daily gift of fresh milk, jellies, blancmanges, and cakes for the Tommies. I am told the same arrangements were carried out in Natal." She specially speaks of the work of the Good Hope Society affiliated to our Red Cross. "The South African ladies made charming holland bags, containing the following articles: pyjama suit, towel, soap, brush, comb, and tooth brush, sponge and bag. Each Tommy in the train received one of these kit bags, also the officers, the only difference for the latter being the addition of a pair of felt slippers." Miss Makins feels that everyone gave most lovingly of their best, and without ostentation, and that their efforts have scarcely received the general recognition they deserve.

### THE SICK AT HOME.

A PLEA was put in by Dr. Chancellor at the annual meeting of the Banbridge District Nursing Society, which should prove irresistible to the good people of that part of Ulster. He observed that the nation had spared no expense in sending surgeons, hospitals, and nurses to South Africa, and he thought it was only fair that "the sick at home should not be neglected." There is not, we hope, any serious danger of such a calamity as this. But it is quite as well that in view of winter and its inevitable privation, which so often result in illness, the public, whether in Ireland or any other part of the United Kingdom, should be reminded of their duty to the suffering at their own doors.

### OLD NURSES' UNION IN THE NEW WORLD.

EVERY quarter past and present pupils of the "Prince Alfred Hospital Training School for Nurses" meet in the Nurses' Home of the Old Nurses' Union to keep in touch with each other, and to exchange ideas and methods of work. It is hoped that a cordial feeling of friendship will be promoted by these gatherings and mutual good result from the union of old and new under the wing of their Alma Mater. Papers are read and friendly criticism and discussion encouraged, light refreshments served, friend meets friend, and many a broken tie is mended, many a fading memory revived under the genial auspices of this little union, which is promoted and managed by the resident Sisters, who act as hon. secretary and treasurer. Miss McGahey, the matron, is president, and renders valuable assistance in making the meetings attractive and helpful to all.

### NURSES AND PRAYERS.

THE Camberwell Board of Guardians have rescinded a resolution which stipulated that the appointment of nurses should be contingent on their willingness to publicly recite in the wards a form of prayer arranged by the chaplain. In the course of the discussion which arose on the motion for the rescission, one of the guardians stated that a Roman Catholic nurse had refused to hold her appointment because she objected to saying prayers which were in the Church of England form; and it is impossible not to sympathise with her. Moreover, there is force in the contention that if she had consented to recite the form, it would, in such circumstances, have been only a mockery. We look at the question from the nurse's point of view, but in the interests of religion itself, it is better that there should not be the semblance of a religious test imposed upon nurses seeking appointments at the poor law infirmaries.

### VICTORIA INFIRMARY, GLASGOW.

A VERY large gathering of ladies and gentlemen took place last week, the occasion being the opening of the New Wing of the Home for Nurses at the Victoria Infirmary, Glasgow. The company assembled in the Board room, and the Chair was taken by ex-Baillie Laing, who introduced Mr. A. Cameron Corbett, M.P. The known sympathy of Mr. Corbett with nurses made him an appropriate speaker on the subject of nurses and their work. After the speeches and votes of thanks the company proceeded to the Nurses' Home, where, in a few well chosen words, Mr. Corbett declared the New Wing open. The visitors were then shown over the building, and on all hands one heard most complimentary remarks as to the comfortable and attractive appearance of the Home. Tea was served in the nurses' sitting room, where music was performed during the afternoon by members of the medical and nursing staffs. The addition consists of twenty-eight bedrooms, two sitting-rooms, four bathrooms, lavatories and pantries, and a well-lighted boxroom. This completes the accommodation for fifty nurses, the assistant matron, and night superintendent.

### LECTURES BY "PRO.'S."

LAST week a Lancashire nurse suggested each probationer in a hospital should take it in turns to give supplemental lectures to the others on whatever subject she has been studying or is interested in. "An Old Nurse," who says that she is much interested in the proposal, points out how easily mistakes might, by adopting it, be ground into the minds of the "Pro.'s," and that the little knowledge which the "Pro.'s" have acquired might thus tend to become a dangerous thing. She proceeds:—

May I suggest—what has often struck me—that the doctors' lectures in hospital might be supplemented by lectures given by old trained nurses? These lectures might take the form of talks on nursing subjects, when the "Pro.'s" could ask about what they did not understand, and discuss difficulties. Would it not be possible that such lectures should be given—not by the busy Matron or Sisters of the hospital, but by those who were trained within its walls but have left, and who often have leisure enough, and love enough of their old hospital, to be glad to undertake some of the work of teaching a younger generation? Such "talks" as I mean would help the "Pro.'s" to understand the doctors' lectures, and be of far more practical use to them than if they gave each other lectures out of their immature and necessarily imperfect knowledge.

### THE TRAINING AT AN AMERICAN COTTAGE HOSPITAL.

THE Portsmouth Cottage Hospital, of Portsmouth, U.S., is said to be a much appreciated institution. It has a course of training for nurses, a feature of which is that applicants are received at an earlier age than is usual with regular schools in America. After the month of probation pupils are required to sign an agreement to remain two years at a salary of ten dollars per month the first year, and fourteen dollars the second. A course of instruction is given by the superintendent, and lectures, demonstrations, and examinations by the medical staff. After being, as the superintendent considers, sufficiently trained, nurses are sent to private cases. The charge made for their services is from ten to fifteen dollars per week, travelling and laundry expenses being paid in addition. This is a high rate for nurses who have not even completed two years' training.

### THE PAY OF NEW SOUTH WALES PLAGUE NURSES.

THE recently formed Australasian Trained Nurses' Association will, it is hoped, be able to secure a higher rate of payment for nurses. Complaints have long been rife in Sydney of the scanty remuneration offered, and the point is driven home by an incident which happened when the plague was at its worst in the New South Wales capital. Owing to the risks of contagion labourers were paid £1 a day for cleaning infected areas, while nurses working at the quarantine station received the munificent sum of 1s. 9d. It appears that there were plenty of brave women available, but that was only because they thought it to be their duty not to refuse.

### THE QUESTION OF RATIONS.

AN effort to secure for two nurses at the Bangor Workhouse Infirmary an allowance of 8s. 6d. a week in lieu of rations, has not been successful. The Local Government Board, to whom an application was made, replied that the Department did not favour any arrangement under which workhouse officials received payments partly or wholly in lieu of their rations, and we concur in their view of the question. But the difficulty of the nurses at Bangor, who complained that they could not have their meals at the same time as the rest of the officials in the house, the hour being often awkward for them, is to be met by supplying them with their rations in bulk for a month. This, of course, leaves it open to the nurses to feast for the first fortnight and starve for the last, a course which is not, however, likely to be pursued.

### "A BOOK FOR YOU, NURSE."

A RECENTLY appointed superintendent nurse at a workhouse infirmary in East Anglia sends us the following:—

On Thursday last I was in one of my wards attending to a little child who was dying of meningitis, when two visitors appeared. These ladies are of the old-young species, got up regardless of expense, and with a great air of patronage. They seemed interested in my poor baby, and asked a great many questions, which I tried to answer in as non-technical a way as possible. They spent some little time in the ward, talking to the patients, and on departing one of them pressed a tract in my hand entitled *The Future of the Lost*, saying as she did so, "There's a book for you, nurse, as I daresay you can read" ! ! ! When they were fairly gone I could not resist going into a long fit of laughter, to everybody's surprise, and I began to wonder if I looked as though I was "lost" or even as if I couldn't read. Methinks I'll have my last Cambridge certificate framed, and hung in some prominent place, as a sort of passport to respectability.

### A VISITING NURSES' ASSOCIATION.

It appears that, under the title of "The Visiting Nurses' Association for Paying Patients," it is proposed to start an organisation "for the purpose of supplying nurses for those who have not the necessary conveniences to have one to live in the house or flat, and also for those who cannot afford the ordinary two-guinea fee." In the "preliminary notice," which has been forwarded to us, it is stated that the fees will range "from 2s. 6d. for an ordinary simple medical case, the visit not to exceed two hours, up to 15s. 6d. for operations and accouchement cases; these fees to be regulated, as far as possible, by the circumstances of the patient." In special cases, we understand, it is in contemplation to arrange an inclusive weekly fee ranging from 12s. 6d. The scale of fees is reasonable, but the Association will, of course, need a representative committee to enable it to appeal with confidence to the public.

### A QUESTION FOR THE NEXT LONDON SCHOOL BOARD.

It is possible that the London School Board which is to be elected next month will determine to adopt the suggestion of Miss Honnor Morten, and have nursing taught at the evening schools by trained nurses instead of by doctors. At her instance, the point has been referred to the Evening Schools Committee of the old Board. Miss Morten contends that the lectures were formerly very much better given by nurses at 10s. a night than they have lately been given by doctors at a guinea. Naturally, trained nurses agree with her.

### ENGLISH NURSES AT WEI-HAI-WEI.

There are English, as well as American nurses at the front in China; for we learn that Nurses Barr and Batchelor have been sent from the Government Civil Hospital at Hong Kong to the base hospital at Wei-Hai-Wei, and have their hands full.

### THE CHILDREN'S HOME.

In the interesting annual letter issued by the Sister and Miss Hoskier, the honorary secretary of the Children's Home at Much Hadham, Herts, it is stated that during the past year little convalescents were sent from eight London hospitals—namely, 27 from the London, 18 from the Mildmay Mission Hospital, 13 from the Evelina Hospital for Children, 6 from King's College, 4 from the East London Hospital for Children, 3 from St. Mary's Hospital, Plaistow, 2 from Queen Charlotte's, and 1 from St. Bartholomew's. These figures attest the confidence which is placed in those who are in charge of the children, and the value of the work which is being done. We hope, therefore, that the appeal for financial help with the view of liquidating a deficiency of £260 will be successful.

### NURSING AT CROMER.

At the Cromer Cottage Hospital, a picturesque gabled house, work of an ever-increasing importance is being carried on. Though the institution has only 10 beds, the nursing has become such as one does not usually find in a "cottage" hospital. The matron—Miss Brooks, from the London Hospital—who has been there five and a half years, is assisted by Miss Battersby—also a "Londoner"—and two probationers. The cases are very varied, and the probationers find that the work affords them an excellent preliminary training. It is expected that before very long a larger hospital will be built. Then, of course, the staff will be augmented, and perhaps the matron will take paying probationers.

### A SCHOOL FOR COLOURED NURSES.

There has just been opened at Greenville, in the United States, under the auspices of Dr. Eliza Grier, a school for coloured nurses. The period of training is two years, and instruction is given by means of lectures, recitations, demonstrations, and chemical teaching. One of the rules of the school is that applicants for training must not "be less than eighteen years of age." This means that at twenty a young woman of colour, if she has received two years' training, is qualified to nurse coloured people.

### BATH ROYAL UNITED HOSPITAL.

The final examination for third-year nurses was held last week. Seven nurses presented themselves for examination, all of whom passed successfully. Nurse Rose Kimber, who wrote excellent papers, was awarded the gold medal, and Nurse Ida Nash, whose paper was next in order of merit, received the silver medal—both the gift of the President. The examiners for the year were selected from the senior staff of the hospital.

### SHORT ITEMS.

"St. Andrew's House" Hotel and Club for Nurses Mortimer Street, W., will be inaugurated on Saturday, when there will be a reception from 3 to 6.—At the second annual meeting of the Ahoghill, Gracehill, and Galgorm Nursing Society a very satisfactory report was presented, showing the organisation to be in a sound financial position, and a balance on the right side at the bank.—A meeting of the General Council of the Royal British Nurses' Association will be held at the rooms of the Medical Society, 11 Chandos Street, Cavendish Square, on Friday, October 26th, 1900, at 5 p.m.—The Countess of Meath presided at a meeting of the Meath Workhouse Nursing Association on Monday last. Miss Annie Lee, hon. secretary, reported that since the last meeting of the committee in July assistant nurses had been provided for the workhouses at Plymouth, Fulham, Wheatenhurst, Maidenhead, Newton Abbot, Knutsford, and Hastings; that twelve candidates had been accepted as probationers, and placed at the Cheltenham Hospital; the Crumpsall Infirmary; St. Luke's Hospital, Halifax; St. Peter's, Kilburn; and Woking, Mosley Hall, Birmingham, Colchester Hospital, and Mildmay Memorial Hospital.—An interesting course of lectures on "Hygiene and Human Physiology" is being given at the Westbourne Park Institute, every Monday evening, from 7.25 to 9.30 p.m., by Mr. Arnold Maddox. One nurse who attended the class last winter passed successfully the examination of the Society of Science and Art, and has now a lucrative appointment as matron in a Colonial hospital. The fees are moderate.

### Lectures on Medicine to Nurses.

By H. A. LATIMER, M.D. (Dunelm), M.R.C.S. Eng., L.S.A.Lond.; Consulting Surgeon, Swansea Hospital; Past President of the South Wales and Monmouthshire Branch of Brit. Med. Assoc.; Past President of the Swansea Medical Society; Hon. Life Member of the St. John Ambulance Association, &c.

THE PULSE—(*continued*).

CONTINUING to speak on the examination of the pulse—a matter with which I was dealing when interrupted in my last lecture—I think it is probable that you would be glad to have an explanation of the reasons it is so carefully investigated, and the lessons which we learn from doing so. The frequency and rhythm are indices of similar actions in the heart itself; for if this "willing slave," as one writer has called it, does not do its work with due regularity, or if it contracts a few times or many times to the minute, the pulse-wave will at once reveal the fact. And we learn even more than this fact by the examination, for we can differentiate between an irregularity caused by an inherent disease of the heart itself and an irregularity caused by some disturbance of its action through the intervention of the nervous system, which is the controlling and governing agent of this as well as of all other functions in the body. Thus we distinguish between changes of rhythm of an abrupt character which are caused by perturbations of the nerve-control over the action of the heart and the changes of rhythm and of volume in the pulse-wave which result from a faulty action in the heart due to disease in its muscular structure. The contractions or dilatations of the walls of the organ are in response to nerve influence carried to it through threads of sympathetic nerves connected together by little masses of nerve-matter which are called ganglia (from a Greek word meaning a knot), and a controlling action is exercised by a great nerve, called the pneumogastric nerve, which is a part of the brain-and-spinal-cord nervous system. This pneumogastric nerve is called by some the pendulum of the heart, on account of the power it exercises over that organ. When its influence is withdrawn from this region, the action of the heart is prone to be rapid or faulty in regularity; when it acts in vigour, and an excessive nerve influence is exhibited, it restrains the heart with such force that its action is slowed down, and the pulse becomes correspondingly slower than its normal rate.

Again, I have spoken of the volume and resistance of the pulse, and it is desirable that I should tell you what significance these points bear to the physician. This part of the subject comes under the heading of "tension" of the arteries, and is one which bears greatly upon the general state of health of the patient, as well as upon the subject of the healthy condition of the vessels themselves. For when the nervous system is relaxed as a result of a want of general tone, the nerve influence exercised over the vessels, through the fine strands of sympathetic-nerve fibres which are everywhere distributed over them, participates in the general relaxation, and the vessel-walls become slack and yield readily to the blood-waves which are distending them, so causing a pulse of large volume, which can be easily compressed and obliterated by the finger placed upon it—the so-called pulse of low tension; when an irritating condition prevails, the vessels respond to a stimulation from the same nerves, and their walls become tense and resistant, and the blood-wave within them becomes smaller in volume and more forcible in character—the so-called pulse of high tension. It is only by the thoughtful handling of many pulses that the finer gradations of these different conditions can be accurately gauged. It takes some time and study to learn all about a pulse of high tension, for the resistant state of the artery is not so much a matter of the way in which the pulse-wave beats against the controlling finger of the examiner, as of the complete emptying of the vessel between one wave and its successor. I need hardly tell you that a pulse of persistently high tension is of great import. It forms a special feature in some varieties of disease of the kidneys, when the smaller blood-vessels throughout the body become contracted, and it is also a frequent condition in old age. When it obtains, the heart has to do its work with more than usual difficulty, and has to contract with extra vigour so as to overcome the obstruction to the flow of blood all over the body. If there is a weak point anywhere in one of the vessels this is apt to give way under the extra pressure exercised upon it by the strong impact of forcibly driven waves of blood, and a breakage occurs; then blood escapes from the ruptured artery and an internal hæmorrhage takes place. Thus we meet with attacks of apoplexy where vessels have ruptured in the brain, and with hæmorrhages in the spleen, kidneys, and elsewhere. So a pulse of high tension gives us a warning of impending mischief, just as the rise and fall of barometric pressure do to sailors and managers of coal mines. On meeting with it we immediately seek for a cause of its existence, and endeavour by all means within our power to remove the mischief which lies at its back. Arteries, like other structures in the body, are liable to undergo degenerations, and, besides a liability to their walls becoming rigid and brittle through the deposition into them of earthy lime salts, they are peculiarly liable to suffer from a change which is known by the name of atheroma. In atheroma fatty degeneration occurs, pus appears, ulcers form in the walls of the arteries, which then yield to internal pressure, and so give rise to those formidable tumours called aneurisms, or the vessels break and bleedings occur. It is sometimes possible to detect the calcareous or earthy degeneration, when it takes place, by the peculiar feel of the artery under examination as it is rolled beneath the finger. Hence the movement of this kind which you will often see the physician make when his finger is on a patient's wrist. Before discussing the subject let me mention that there is an instrument, called a sphygmograph, for making tracings of the pulse. It consists of a lever and a clock-work mechanism for ensuring the passage of prepared paper, on which impressions made by the former are recorded. One end of the lever lies, with a definitely arranged pressure, on the artery which is being examined, and the dilatations and contractions of the vessel are communicated to a needle tracing-point, and from thence to the paper, in such a manner that anyone who is skilled in this work can read the signification of the message left there, just as anyone can read the messages printed on a tape from a telegraphic machine. A sphygmographic tracing is one of the most interesting records that one can see, provided its signification is understood. It reveals in the most graphic manner the whole working of the heart and arteries—from the time that the blood-stream is propelled from the former, until it reaches the recording machine; and by the lines of ascent and of descent, and by the interruptions in these curved markings, it can be seen with what vigour the fluid was ejected from the heart, how the vessels dilated to allow it to pass, how the aortic valves shut to prevent a reflux of blood to the left ventricle, and how the vessels vibrate during the processes which are going on within them. Another matter which you will do well to make an especial point of regarding is the colour and characteristics of the face as indications of disease. If you cultivate the habit of observation in this matter as in all others, it will stand you in good stead throughout life, nothing being more true than the fact that by paying attention to details the big concerns of life will be made easy to you: they take care of themselves.

### Nursing at Pinetown Bridge, Natal.

A MEMBER of the Army Nursing Service Reserve, who was attached to the Princess Christian Hospital at Pinetown Bridge until early last month, sends us the following interesting experiences:—

My first ward consisted of 18 beds, and on April 9th the Princess Christian Hospital train brought a load of poor Tommies, most of them very ill with either enteric or dysentery, and, in spite of all care, very exhausted by the long hot journey down country. Words fail to describe some of the poor fellows' condition, many of whom had been lying for days, if not for weeks, on a waterproof sheet on the sand in a tent, where water was scarce even for drinking, and not to be had at any price for washing purposes. One man, I remember, told me it was the very first time he had slept in a bed since coming out in November last. Many were quite delirious, constantly getting in and out of bed, and my only helpers were the orderlies, who did their very best, but, alas! knew so little how to nurse a really sick man.

### Eighteen Acute Cases.

My heart sank as I looked round my 18 beds, and thought of all my four hours' charts, spongings, drink every two hours or oftener, to say nothing of medicines and washings, and the daily routine of ward work. Let any Sister in England imagine 18 very acute cases sent into her ward in one half-hour's space—wanting everything to be done for them—and, instead of a full staff of trained nurses, two men, who knew a little theoretically, but had never really nursed before, and she can understand my feelings. Still, all things come to an end. My two doctors were awfully good to me, and the orderlies obeyed without asking why, learning very quickly by copying me, for I had no time at first to teach them; and after three days' working from 6 A.M. to 10 P.M., with just the break for meals, we had time to breathe and to find that our patients were really improving. I am thankful to say we pulled all through, though nine of them seemed at first hopeless. How proud we felt when all in our ward were pronounced by their doctor to be "out of the wood," and instead of parched lips moaning out incoherent words or fighting over again the awful strain of Spion Kop or Colenso, we had grateful words and glances, and it was, "Now, Sister, you let me just wash my own face and hands this morning, then you'll get No. 17 done sooner," or "Here, Sister, I can take my temperature to-day; I won't let the 'glass' fall, I promise!"

It was wonderful how soon the rest and pure air made different men of them, and both to patients and nurses it was a delight when we could carry them, "*à la* Banbury chair," between two orderlies, to a shady place outside the ward.

### The First Batch of Convalescents.

The first batch of convalescents we sent home caused immense excitement. All my special men were to go, so of course I had to take all their photos (having a pocket folding Kodak) in a group, with doctors and orderlies, and again individually; but when they were ready for the major's final inspection, blue hospital suits discarded, and the beloved khaki uniform once more on, it was pathetic to see how loose the coats hung, and what white drawn faces the helmets shaded, while here and there one saw an empty sleeve or a crutch, and more than one eye with a shade that meant the sight had gone.

### Seeing them Off.

Nearly all the staff went to see them off at Pinetown Bridge Station, and what a cheerful company they were—so grateful to doctor and Sister for their care, tears in many brave eyes as they said how thankful they were to be going home safe again! Tommy may not be religious, but several of the men told me that this war had made them realise at last that life here was not all, and that the fact of seeing men and women ready to work (and die if needs be) to help them had brought home a sure conviction that they served a God of Love, who had guarded Tommy as he did his duty to his country.

### The Queen's Chocolate.

I wish our Queen could have seen the joy and pride that her chocolate gave some of them. "Eat it, Sister?" said one. "Why, no; but I got it safe 'ere"—slapping his breast-coat pocket—"and I guess the missus and kids ull take care on it." One poor man confessed with great contrition that he had sold his for 2s. 6d., but he would have given £1 to get it back. Another patient, knowing how much I wanted to get a box, brought his, and offered me it. "You pulled me through the fever, Sister, and I'd not be going home this day but for your care, so take it—do!" But Sister could not accept such an evident sacrifice, much as she appreciated his generosity. As the train moved off I took snapshots, and so we said "Good-bye," amid waving of helmets and handkerchiefs, quite sorry to lose such hearty friends.

### Nursing Officers.

My *next* ward was the officers'—and there I found the work very heavy, but quite a change. My worst case was a very bad one of enteric, an officer who had gone all through the Ladysmith siege, and then caught fever as he was leaving the town. Poor fellow! he had no idea at first how ill he was, and was always so anxious to save me trouble, talking of going home to his wife—they had only been married a few months. He had a bad turn one terribly hot night, and no medical skill or nursing could avail. Happily, he was quite unconscious and free from pain at the last. Then I had one fussy and trying patient, who was not very ill, but resented anyone but himself being seen to. Once I had just gone to dinner when I was hurriedly called back by an orderly. Off I rushed, and found the patient lying back in his easy chair, eyes closed, mouth open, groans of a heart-rending kind filling the air, an orderly standing by fanning him vigorously with one hand, a glass of water in the other. It seemed that after I had gone he had insisted on having ice in his soda and brandy, with the inevitable result that he had a bad attack of spasms, and sent for the doctor and myself under the firm impression he was dying!

### A Real Hero.

Then I had the pleasure of nursing a real hero, who, at Spion Kop, when his captain was shot early in the day, took the lead, led his men up the hill, encouraging, cheering, helping, utterly oblivious of shot and shell all round him, fighting on, as one of his men told me, "Like a quiet little devil, who warn't to be done anyhow!" He came to us with very bad enteric, but youth and his quiet nature pulled him through; and he had been in hospital for more than a month before I found, quite accidentally, that he had three Boer bullets still in his side from Spion Kop. His shy nature had a great shock the first day he was carried outside the ward into the fresh air, for several of the men in his company found it out and greeted him with hearty cheers. He looked absolutely as if he wished the ground would open and swallow him up, chair and all!

### The Night Duty.

I really enjoyed my time on night duty; it was worth the hot sleepless days to see the sun rise. During my night duty a huge horned owl took to following me as I went round, so I coaxed him with bits of raw meat, and he finally got so tame that he would come at my whistle, and eat out of my hand. I never saw a bigger bird. He would sit on a post in the avenue and stare at me with his great yellow eyes, ruffling up his feathers if I went too near him. I am fond of pets, so had a monkey (who ran away), several cats, a mongrel dog whom we made a member of St. John's Ambulance, and wore a Red Cross on his collar, and several chameleons, one of whom I had for five months, and brought home to England, where he is quite happy catching flies in my greenhouse at present. But I think of taking him back again, as I doubt whether he could stand an English winter.

### Sketches from the Maratha Plague Hospital, Bombay.

BY ONE OF THE NURSES.

(*Continued from page* 31.)

BÁPPOO—our little favourite of whom I wrote last week—and his mother were beggars. The woman was dying when brought to us. Báppoo was a very devoted son; he could not understand why his mother should be kept on milk diet, and it was pretty to see him rise up and face the doctor on his rounds and demand in a manner at once half-fearful, half-brave that she should be given *dhála bháta* (dhal and rice) to eat. He grieved bitterly when she left him, but was soon first and foremost in all manner of mischief. His principal misdemeanour was that he drank quite half a bottle of cod-liver oil and malt, which had been specially provided for a sick ayah.

### A LITTLE HEROINE.

Little Gungá belonged to the Parbhu caste. Her father was a clever man and well-educated, a clerk by profession, but unfortunately an inveterate drunkard, and in consequence his wife and children suffered severely. Gungá and her mother had been taught by the ladies in the Free Church Mission. The child was remarkably sweet and gentle in all her ways, and of a refined and delicate appearance. Her death was one of real self-sacrifice, for she contracted plague in the wards, where her parents insisted on keeping her in order that she might help in nursing her younger brother and sister. In vain we remonstrated with them. The answer was always the same, "What could they do?" The mother *must* stay with the cherished boy, and Gungá could look after the little sister in the female ward. Fortunately for Gungá her brother was convalescent when she was stricken, so she had the comfort of her mother's love and care. We could see by the woman's tearless eyes and drawn face how much she grieved when the little girl was taken, but all that the father seemed to care for was that the child's grown husband had telegraphed from his far-away home that he would not pay the expenses of the funeral.

### SOME PATHETIC SCENES.

Rámá and Sudáma can hardly be classed amongst the children. Though still very young, they were working for themselves, and when first admitted had no friends with them. Both were seriously ill, but they struggled through the first anxious days, and reached a point when recovery seemed just possible, though hardly probable. Both suffered from chorea and meningitis, and were unconscious for many days. It was pitiable to see their restlessness, and to hear Rámá's incessant cry, night and day, "*Ái, ata mi kaya karu?*" (Mother, now what shall I do?) answered from the other side of the ward by Sudáma's perpetual "*Age bayo!*" (Oh, dear me!) One afternoon Rámá's mother arrived from Poona, a poor, gaunt, ugly woman, but with a true mother's heart beneath her faded *lugada*. Fearing lest the sight of her should be too much of a shock for the lad in his weak condition, we bade her wait a minute while we tried to prepare him for her coming. "Rámá, would you like to see your mother?" "*Hoya! hoya!*" (Yes! yes!) in a fretful tone. "Well, Rámá, your mother has come"; and we drew her gently forward and bade her speak to him. It was a pretty sight to see her place her hand upon his head and bend down to kiss the poor wreck of a boy again and again. From that time he began to recover; we heard the weary cry no longer, and though still very restless and irritable, he struggled on towards convalescence. At last he was well enough to leave us. The travelling expenses of the happy pair were paid out of the fund provided by Government for that purpose, and they left for Poona with many expressions of gratitude and most profound salaams.

### A SADDER ENDING.

Sudáma's story had a sad ending. His mother came to Bombay on hearing of his illness. She fed and tended him most assiduously, but Sudáma grew worse and worse; he lay on his cot like a crushed heap, his body contorted and twisted with painful convulsions. His sufferings were intensified by the belief that he was possessed of an evil spirit, which he used piteously to beg might be exorcised. The steam bath was tried, and soothed him for a time, especially as the young Hindu doctor in charge of it, moved with pity for the boy's miserable condition, told him to fret no more about the devil because it had been driven out of him by the steam. In a few days, however, all the worst symptoms and wretched fancies returned, and it was clear that death was inevitable. "*Sudámáchi ái ápalá tónda Sudámacháya tondálá lávita áhe*" (Sudána's mother is putting her mouth on his mouth) said a ward boy to us with a shocked look on his face. We turned, and it was as he had said. The broken-hearted mother lay, her body stretched upon the wasted body of her child. Her warm lips were pressed to his lips, as if she would give him new life with her breath. Those around looked on in silent sympathy. For a native respects a mother's privilege and a mother's grief. But we were obliged to interfere. It was not good for the rest of the ward, nor safe for the mother; so we drew her gently away, and tried to accomplish the sorry task of comforting the comfortless. A day or two after Sudáma died, and we were all thankful that at last the weeks of painful struggle were ended.

### VICTIMS OF ALL AGES.

It would be easy to multiply story upon story, each more pitiful than the other. Here we see a girl-wife, hardly more than fifteen years old, watching, with her mother-in-law, the death-bed of a fine stalwart young man, her husband, and beseeching us to save him because there is a *lahána lahána bála* (little, little baby) at home. In the next bed a strongly-built cooly is painfully struggling for breath. His old mother, squatted at the top of the cot, has made him a pillow with her knees, on which she rests his burning head—oh, how tenderly!—whilst with quivering lips and trembling voice she tells us that he is her last remaining treasure. Here a group of children watch the light fade out of their father's eyes. Mother and the baby went some days ago, and now they are alone. There a grey-haired couple close the eyes of their only child, and with shaking hands endeavour to straighten his little convulsed limbs. Here each member of a family is trying to feed with a spoonful of milk the man who can no longer swallow, fearing lest he should die ere they have time to perform this pious act. There a woman weeps the name of "Ráma" over the brother who, through all the weary years of her widowhood, has protected and supported her. Here a husband turns from the pretty young wife who has lost the sight of both eyes, feeling that her death is no longer a matter for regret. There a woman raises a sharp cry of "*Geli!*" (Gone!), and flings the blanket over the face of her still gasping daughter.

### THE PERSISTENCE OF THE PLAGUE.

Scenes such as these have been enacted over and over again in Bombay for the last five years, and still the plague is with us. We talk of the next epidemic in as matter-of-fact a way as we talk of the next monsoon. Science, skill, money, have each and all been unable to stay its ravages. It stalks through our highways and bye-ways. It lurks alike in the mansions of the rich and in the filthy chawls wherein the poorer classes crowd and huddle together. It chooses its victims without respect of persons. In this house only one is taken, in that only one is left. "All things come to those who wait," says the proverb, and we wait with aching hearts for the end of the visitation.

### The Nurse in Hot Climates.

By EDWARD HENDERSON, M.D., F.R.C.S. Edin., late Surgeon, General Hospital, Shanghai.

(*Continued from page* 29.)

*Punkahs.*—Punkahs are suspended from hooks driven into the ceiling of the room, or, in some private houses, from one of the cross-bars in the framework of a mosquito house, where a house of the kind takes the place of the ordinary mosquito net. In the East they are all pulled by native servants. Whatever material is used in making the body of the punkah, a certain proportion must always be maintained between size and weight. If the punkah is too light, the to-and-fro movement will be jerky and unequal; if too heavy, the coolie who pulls it will be taxed unnecessarily. When punkahs are used during the night, as is often the case in private houses, it is usual to provide two coolies, and the nurse will make what arrangements she can to prevent the service from being interrupted. In very hot weather, if a night punkah stops working, the patient is sure to perspire profusely, and the rapid evaporation which follows when the service is resumed chills the surface of the body in a way better avoided. Nothing more surely disturbs sleep than an irregular punkah service during sleeping hours. Ordinary mosquito nets will need to be removed to make way for a night punkah; and when this is done, protection from mosquitoes must be got by lengthening the frill or flounce which forms the lower border of most punkahs, so as to bring the range of movement within a foot or two of the patient's face. If this is properly managed, and the punkah service is not an interrupted one, mosquitoes will be effectually driven away; the new arrangement may possibly be at first objected to by the patient, but tolerance is soon established. The sudden onset of serious symptoms is one of the features of disease in the tropics. Should it happen that a punkah is unexpectedly needed during the night where no previous provision has been made for its use in the room occupied by the patient, the bed in private houses may need to be removed, or another improvised in one of the sitting-rooms. In the dining-room, where a punkah is always to be found, a mattress laid on the table may do at a pinch; and if mosquitoes are troublesome, the nurse can easily lengthen the punkah frill with the aid of a bedroom towel and a few safety-pins. The clothing of a patient sleeping under a punkah needs special attention, and a thin india gauze singlet will in most cases be found the best covering for the body; a flannel cummerbund, or cholera belt, is a good addition to this. Additional coverings are often needed in the early hours of the morning, and should be provided for by a thin blanket kept in readiness at the foot of the patient's bed. If desired, the loose pyjama shirt may now be put over the thin singlet covering the body. When heat is excessive, the patient may be unable to tolerate any covering, and contact even with the sheet enveloping the mattress is objected to; under these circumstances a piece of the soft grass matting made in many parts of India and China will provide a cool and comfortable resting-place in bed. Children, who often perspire profusely about the head and neck at night in hot weather, may with advantage have their pillows encased in this matting; but great care must be taken to select the proper quality; it must be soft and pliable; the commoner kinds make a very uncomfortable resting-place for the head. In hospital practice it is not always possible to make a sufficient use of punkahs; but the effort should always be made in warm weather to provide one during the hottest hours of the day, and when the principal meals are served in the wards. When punkahs are not to be had, or the service cannot be sufficiently maintained, fans should be given to the patients. Palm-leaf fans are the best to use; they can be got almost everywhere throughout the East, and, as they cost a mere trifle, can be destroyed when they have served their purpose.

*Ice.*—As a result of improved methods of manufacture, and the increased facilities provided for distribution and storage, there are now few places in the East where European nurses are employed where ice cannot be obtained: the purity of the supply is another matter, and one which, unfortunately, can seldom be guaranteed. It is the nurse's duty to ascertain the character of the ice she uses; and if doubtfully pure it should, of course, neither be given to the patient to suck, nor be put in the liquids he drinks. The ice-chest is then its proper place, and, as ice-chests are used almost everywhere where ice can be got, the nurse should have no difficulty in at least securing the cooling of liquids and the preservation of the food she gives to her patients. When ice is of good quality it is sometimes ordered to be crushed, and in that state given to the patient, to be taken by him from time to time as he may feel inclined. In hot weather it is a little difficult to arrange this so that the supply shall remain constant and within the patient's reach. To preserve the ice it should be kept as far as possible covered from the air, and removed from contact with the water it forms in melting; and this is done by putting it when crushed in a flannel bag, which can either be suspended close to and on a level with the patient's bed over a basin on the floor, or be simply laid in one of the basins used on washing-stands to hold the sponge. When ice cannot be obtained, liquids may be cooled by immersing the vessels containing them in one of the wells so often found sunk in the compound surrounding a bungalow in the East. Unfortunately the water in nearly every well of the kind is of doubtful character, and, if recourse is ever had to this plan, care must be taken that no communication takes place between the liquid to be cooled and the water in the well—an accident sometimes difficult to avoid. This is a matter which the nurse should never trust entirely to a native servant. Rapid evaporation from a wetted cloth surrounding the containing vessel will do something towards reduction of temperature, and this plan was frequently taken advantage of on board ship in the old days when the ice supply—a much more precarious matter then than now—ran short; the bottles, covered as described, were hung up by the stewards in an open port on the windward side of the ship.

*Taking Temperature.*—The duty of taking temperatures is one which in most private houses, and in many small hospitals, devolves chiefly on the nurse. There can be little doubt that the temperature of patients needs to be more carefully watched in a hot than in a cold climate; for, besides the cases of sunstroke and heat-apoplexy which are more directly associated with the condition of the atmosphere, fevers of all kinds under the influence of great heat are apt to run a somewhat irregular course, while in the case of those due to malarial poisoning a sudden, and it may be dangerous, rise in body temperature is by no means an uncommon occurrence. Hyperpyrexia, whether it occurs during the course of a specific fever, follows direct exposure to a tropical sun, or results from no other apparent cause than sustained tropical heat complicated it may be by the abuse of alcohol, is a condition attended with danger to life, and one in which active treatment cannot be long delayed.

### Nursing at the Royal Edinburgh Hospital for Sick Children.

A CHAT WITH THE MATRON.

BY A CORRESPONDENT.

I WONDER how the children feel, if ever they come out of the closes of Cowgate, to be nursed in the large, bright, airy wards of their hospital across the Meadows? It is such a contrast from stuffy, ill-ventilated houses—where garments hang from every window to dry, and where almost every second house is an old-clothes shop—into the clean, sweet wards, with their flowers and pretty cots, and the smiling faces of the sisters and nurses; and the nurses here are bound to be smiling ones, for, according to Rule 11, "while impatience, ill-temper, or anger towards the patients will be followed by dismissal, the mere inability to make children happy will of itself be regarded as a sufficient cause for not retaining a nurse in hospital."

THE KING OF SIAM'S DOLL'S-HOUSE.

The King of Siam recognised the value of amusement, for the first thing the Matron showed me was the enormous doll's-house given by him after his visit. "We keep it in the hall," she said, "for the children of visitors to play with when the patients' friends come to see them, as no visiting children are allowed in the wards. It has eighteen rooms, and must have cost at least £50. It takes half a day to spring-clean it."

IN THE WARDS.

From this point we passed up the wide staircases and into the wards. It was dinner-time, and the little patients had their tables across the bars of their cribs, and with their plates of chicken mince, or whatever special diet had been ordered them, were busy eating away; while one of the nurses was standing over a cradle-cot, waiting for the tiny fragile being inside to swallow its milk from the bottle which she held for it.

"Here is an opportunity of seeing how the babies take their medicine," said the Matron; and we watched while the nurse opened the patient's mouth and poured the dose in—it went down without a murmur! "That was nasty, wasn't it?" the Matron observed, patting the little one's head; but no Stoic could have resigned himself with more fortitude to his fate than this infant in his cot.

"Is there an age limit for the admission of patients?" I inquired.

"The general rule is not to admit under two years old," she replied; "but the directors have relaxed this, and now no child is too young or too helpless to have the benefit of skilled nursing and care. This has caused us to increase our nursing staff, as we have frequently from twenty-five to thirty babies under a year old; and the careful feeding of these takes up so much of the nurses' time."

"How many patients had you last year?"

"One thousand four hundred and fifty-six in the wards, and over six thousand were treated as out-patients."

THE TRAINING.

"What is the period of training?"

"Probationers are received for three years, but candidates must submit to a three months' novitiate, at the end of which, if they have proved unsuitable, they may be dismissed."

"Does this often happen?"

"They are not often dismissed, but they sometimes leave, having found the work quite different to what they expected, and wishing more liberty for outside interests and pursuits."

"How about instruction?"

"Lectures are given on anatomy and physiology, surgical and medical nursing, nursing hygiene, ward administration and management, by the honorary visiting staff, lady superintendent and sisters; and there is a demonstration by the honorary electrician, and an examination after each course."

"At what age are probationers admitted?"

"From 21 to 30 is the age limit, but we seldom take anyone over 24 or 25, as they are then eligible for adult training, and encouraged to take it."

HOURS AND HOLIDAYS.

"What hours do the nurses work?"

"Nurses and probationers go into the wards at 7; the sisters half an hour later. Sisters and nurses are off duty from 2 to 3, or 3 to 6·30, alternate days—the nurse, of course, being on when the sister is off; and probationers have two hours off duty every afternoon. Supper is at 8.10, prayers at 8.40, and lights are out by half-past ten."

"What about holidays?"

"The sisters have one month in the year, and once a fortnight from Saturday afternoon to Monday morning; nurses have three weeks and two days yearly, and from Saturday afternoon at 3 P.M. to Sunday night once a fortnight; probationers three weeks yearly, and a whole day once a month. One of our difficulties is with probationers who go for their holiday, and don't return on the appointed day."

"But is there no rule about this?"

"Certainly there is—a very strict one; but as long as there is nothing legally binding about nurses' agreements, so long the moral obligation will be disregarded by a certain class of girls who have not taken up the work in a proper spirit, and do not wish to spend the third year with children, having tired of them, or for various other reasons."

THE NURSES' ROOMS.

Having gone through the wards, and visited the children lying out in the sunny balconies, we looked into the sisters' and nurses' sitting- and dining-rooms. "The nurses have the best of it here," said the sister who was with me at this point, the Matron having been called away; "their sitting-room looks more homely than ours, I always think." But to the visitor both looked very bright and cheerful, with piano and flowers, and comfortable chairs, and pictures.

"You have quantities of flowers," I remarked.

"There has just been a flower service in Edinburgh," she replied, "so we have more than usual," opening as she spoke the door of the maids' room, which also had its piano and easy-chairs, showing the recognition on the part of the directors that the hospital servants form an integral part of the staff of the institution.

"Most of the nurses are natives of Scotland, are they not?" I asked when the Matron returned.

"Yes, but we have some natives of England," she replied, as she led the way to the nurses' bedrooms, and opened the doors for me to peep in. "We have been waiting for the plaster to dry before painting; but when our walls are properly coloured, we shall be much more strict about holes. One of the rules says that no nails are to be driven in, but no rules can make a room look tidy when the walls are like this."

They were of a dingy, nondescript colour, that clashed with everything; but even here one could see, as the matron said, the difference between a tidy nurse and an untidy one: bedrooms are tell-tale places indeed, and one or two looked quite pretty. "As we are very short of sleeping accommodation at present," she went on, "there is some excuse for the nurses, their rooms having to be occupied during their holidays by others, and frequently changed; this will soon be altered, as we have purchased the two adjoining houses, and when the necessary reconstructions have taken place we shall have much more comfortable quarters for them."

THE THEATRE.

We visited next the surgical theatre, where a nurse was cleaning up after an operation; and the Matron displayed the glories of the new instrument cases, the electro-plated steriliser, and glass-topped operating-tables.

"This is one of the most important medical schools in Europe," she said; "so, naturally, we wish to have everything up to date. During 1899 we had 136 students here, male and female. The theatre compares favourably with any in the kingdom, and since its completion, a little over a year ago, the work of the surgeons and nurses has been greatly facilitated. We have a resident lady dispenser, whose work is much appreciated by the doctors. One of our needs is a larger laundry. The present one does not at all satisfy our requirements. In a children's hospital there is such constant daily washing; and we wash, as well as provide, all the children's clothes. This department is to be enlarged, the work being already in hand. In the out-patients' department the provision is again quite inadequate. Our patients frequently come long distances, and there ought to be more comforts for the mothers while they wait their turn with the little ones. The whole building is too small, and ventilation bad; but it is to be enlarged shortly and improved."

"Do you receive accidents?"

"Not many; they generally go to the Royal Hospital."

UNDERGROUND.

The utmost possible use is being made of the cellars underground, and the Matron exhibited with a motherly pride the brush-room, carpenter's shop—where the repairs of furniture, electric light, &c., are done on the premises—store-rooms for crockery, &c. &c.

"It's not complete yet," she said, in a hopeful tone; "but we are going on, and shall get things straight in time."

"These rooms are your own idea?" I inquired.

"Yes; a very great deal of organisation rests with the matron, as there is no medical superintendent, or resident secretary, or steward. The resident medical and surgical officers change every six months; this leaves the practical organisation of the work of the hospital to a large extent in the hands of the matron and her assistant, who, in addition, have the superintendence of thirty-six nurses, twenty female servants and five male ditto, the housekeeping and the actual nursing of any sick members of the staff, which keeps their time fully occupied."

THE MORTUARY.

"We have the advantage in Edinburgh of a society called the Social Union; it is composed of the ladies who collect rents on slum property, and the commissions paid them by the landlords are put into a fund for bringing beauty home to the poor. That is how it comes about that our children's mortuary is so beautifully decorated; this fund paid £20 for the materials. But the decorations themselves are done for love; they are the work of Mrs. Traquair, the wife of a doctor here, and have been removed to this building from the old mortuary in Lauriston Lane. A great deal of the work had to be restored after removal, and much of it is new."

It would take too long to describe these beautiful mural decorations, for they are full of symbolism; a deep religious tone pervades them, and the faces of many of the spirit-children are those of little ones in the hospital whom the matron has known.

The beautifully hem-stitched sheets and altar-coverings are the work of Edinburgh ladies.

THE FAIRY GODMOTHERS.

"I cannot say too much about the kindness of our Ladies' Committee," remarked the Matron, as we returned to her sitting-room. "They provide us with flowers, and give the children so many treats in the way of drives, picnics, birthday parties, *et cetera.* Our Ladies' Work Guild supplies most of the children's clothes, a very large item in a hospital of 120 beds, and where the patients nearly all come from poor homes. They also give many little comforts for the nursing and domestic staff."

### Presentations.

ST. SAVIOUR'S INFIRMARY, EAST DULWICH GROVE.—Miss Williams, who has held the post of Sister at this infirmary for ten years, was presented on the 10th inst. by the matron, medical and nursing staff with a handsomely fitted tea and lunch basket, framed engravings, and books, accompanied by many expressions of regret at her resignation.

REDRUTH DISTRICT NURSING ASSOCIATION.—As a mark of appreciation of faithful and valuable services to the sick of Redruth during the past six years Miss Brindley, the district nurse, has just been presented with a Queen Anne solid silver tea service with her monogram, "E. S. B.," engraved on each piece, and a cheque for a substantial amount, representing the balance of contributions received. Miss Brindley, who after more than twenty years engaged in nursing work (including six years at Redruth) intends resigning active work, will probably spend the winter in the South of France. She carries with her many good wishes from residents in her last sphere of labour. This public testimonial to the value of their nurses' services is a source of gratification to the Redruth District Nursing Association.

CITY HOSPITAL, BRADFORD.—Nurse Page was the recipient of a handsome afternoon tea service from the nursing staff of the City Hospital, Bradford, on her leaving to take over her new appointment at the Sanatorium, Morecambe.

BANGOR NURSING INSTITUTE.—Nurses Dean and Bettany have been the recipients of a testimonial, the former receiving a writing table and an illuminated address, and the latter a gold watch and chain and a similar address. The testimonial was largely subscribed to by the friends and old patients of the two nurses, with whom they have always been exceedingly popular.

### Minor Appointments.

CITY ISOLATION HOSPITAL, NOTTINGHAM.—Miss Alice Rayner has been appointed sister. She was trained at Halifax (St. Luke's) Hospital and the hospitals of the Metropolitan Asylums Board, and has since been charge nurse at the City Hospital, Bradford, and nurse matron at Braintree.

MONKWEARMOUTH AND SOUTHWICK HOSPITAL, SUNDERLAND.—Miss Gertrude M. Savage has been appointed staff nurse. She was trained at Gravesend General Hospital, and has since been engaged in district nursing at Northfleet; nurse at the Hertford British Hospital, Paris; and the Accident Hospital, Poplar.

MOORFIELDS ROYAL OPHTHALMIC HOSPITAL.—Miss Annie Powis has been appointed night sister. She was trained at Bristol General Hospital, and has since been charge nurse at Victoria Park Hospital, N.E., and sister at the Birmingham and Midland Eye Hospital.

STROUD HOSPITAL.—Miss Bessie Trewin has been appointed sister. She was trained for three years at the Royal County Hospital, Guildford, where she was afterwards, for six months, sister of children's ward. She has since been Sister at Ramsgate General Hospital.

### Our Convalescent Fund.

WE acknowledge, with many thanks, the receipt of a postal note for 5s. from Nurse Groom, Upper Norwood, to our Convalescent Fund.

### Wants and Workers.

NURSE E. D. KELSEY, Ledbury, Herefordshire, would be very grateful for contributions of old rag and flannel for her district.

### Echoes from the Outside World.

### AN OPEN LETTER TO A HOSPITAL NURSE.

THERE have been many false rumours about the betrothal of the Queen of Holland, but on Wednesday an official announcement was made of her approaching marriage to Duke Henry of Mecklenburg-Schwerin. I am sure that all nurses will wish the young sovereign every possible happiness. She has taken time to make her choice, and though she is still only twenty it may fairly be assumed that she has found in Duke Henry, who is just four years her senior, all the qualities which she is satisfied are needed in the man who is to possess her hand and share her throne.

THE fact that for some time past the health of the Empress Frederick has been known to be unsatisfactory naturally prepared us to receive the news of the august lady's illness with apprehension, which was not lessened by hearing of two doctors and four nurses in attendance. From later intimations it would seem that there is at least no immediate cause for alarm, though the bulletin issued on Monday and signed by Professor Renvers and Dr. Spielhagen leads one to dread the recurrence of heart weakness, which, followed by secondary catarrh of the lungs, gave rise to such grave reports last week. Of the Empress herself little has been seen in England for some years; and though she was very near our hearts at the time of her husband's sad illness, her continued residence abroad has necessarily made us less keenly interested in her than in those members of the Royal Family who live in our midst. But she is the eldest daughter of our beloved Queen, whom she now closely resembles in face and expression, and as such, and because we would fain spare our dear Sovereign additional pain or trouble, we receive with gladness any assurances of there being less cause for anxiety and more ground for hope.

WITH regard to the fashions for the coming winter, I am told that ermine is to be as much worn as ever, but I do not fancy the information is worth repeating to the ordinary nurse. Hospital salaries hardly allow of purchasing this rather expensive trimming—expensive not only in the first instance, but because it keeps clean such a short time that it needs to be continually in the hands of the renovator. But you will like to know that red in various shades will be one of the most popular shades during the cold and dull days ahead of us. For those who are sufficiently bold there is the real scarlet of a soldier's coat which can be subdued by a lavish use of black braid or black fur. Now that our troops are coming home, the patriotic women who are dark enough, or fair enough, to know that such a bright hue is becoming to them, will probably catch the "scarlet fever" rapidly from one another, and red will gleam everywhere. You may have observed that Lady Warwick when she spoke at the Second Annual Meeting of the Lady Warwick Agricultural Association for Women, held at Stafford House the other day, was attired in scarlet. But besides scarlet there are a dozen different shades of red, all of which will be fashionable this year.

As to style, the bolero, which has never really been absent for a long time, is in high favour just now, and although it is a pretty mode of trimming a new dress, it is especially useful in doing up an old one. Dresses which have hard wear are very apt to show it under the arms. A bolero covers the faulty part, and daintily renovates the bodice. A black dress with a new velvet bolero in bright red, vivid green, or brilliant orange velvet, embroidered with narrow black braid or fancy trimming, looks charming and costs very little. Paisley is also a good deal used for the purpose, and if of good quality looks well enough. As to jackets, the newest is the pleated coat, which has evidently been suggested by the pleated skirts so much worn during the summer. The pleats start from a plain yoke—which may be braided, outlined in fur, or otherwise decorated—and are stitched down to within six or seven inches of the bottom. The shape is generally sac, and the coat reaches almost to the knees. Only those who can afford to get rid of them as soon as the fashion is passed should indulge in a pleated coat, because they are sure to be merely a fleeting fancy, and next year they will be painfully passé. The sac is also the favourite "cut" for other jackets; but beware of buying one if you have not a nice flat back, or are inclined to slouch. On anything but a trim, neat figure they are horrid; but those who have learnt how to carry themselves well will find them both becoming and comfortable.

THE London School Board has lately caused an examination to be made of the eyes of those children attending classes, and as a result it was found that out of 338,000 pairs of eyes 79,167 were defective. Of course it must be remembered that twenty years ago short sight and imperfect vision were of no account in the minds of school authorities, a child who could not properly see the blackboard, and so failed to grasp the lesson, being simply reckoned stupid; whilst one who suffered from continuous headache, through straining the eyesight, was considered naturally delicate. Therefore it may be argued that many a boy or girl who would have derived immense benefit from the use of spectacles through ignorance went without, and the number of children incapable of seeing well has not increased in so large a ratio as by appearance it seems to have done. But it is certain that the total of spectacled small mites in the streets is quite extraordinary, and that the percentage of children who require the attention of an oculist is a very large one. There must be some reason for this state of things, and the suggestion of the mischief being due to the imprudent use of perambulators and mail-carts is founded on more than a substratum of truth. In the summer I frequently notice wee babies lying asleep or awake with their faces turned up to the sun, and in the London parks and on the suburban commons the sight of a nurse sitting reading on a seat whilst the baby wriggles about to try and avoid the glare is a most usual sight. The employée of the well-to-do mother errs generally through thoughtlessness and carelessness, not taking the trouble to raise the hood or the sun-cover; the worn-out or cheap "pram" of the poor mother as a rule boasts of no hood which can be used. The result, however, is the same—the child's sight is injured, and frequently for life. This subject should be of especial interest to maternity nurses. If it be possible, in the early days after the baby's arrival, to impress upon the parent the need of shade for the eyes which have so lately opened on the world, a great and important step will have been taken towards improving the eyesight of the coming generation.

I AM pleased to see that the Commissioner of Police for the Metropolis has especially drawn the attention of the small shopkeepers who sell fireworks to the Act of Parliament which forbids them to sell any explosive to a child apparently less than thirteen years of age under a penalty of £5 for each offence. Sir Edward Bradford goes on to say that the police have strict orders to proceed against any persons offending, and it is to be hoped that they will do so, for the small boys of eight or nine who are to be seen at this time of year throwing crackers and squibs at the passers-by, so as to enjoy the fun of seeing them start, are sources of danger, not only to others, but to themselves, and even the most unobservant of shopkeepers cannot fail to be conscious of the extreme youth of many of the offenders. I suppose, notwithstanding all precautions, a few so-called "good-natured" folks will be found who will go and make the purchase for the young brother or the neighbour's child, and thus provide him with the forbidden toy; but that sin cannot be laid to the charge of the authorities—they can only do their wisest and best to prevent accidents.

### Crisis at Croydon Infirmary.

THERE are serious differences between the Croydon Board of Guardians and the matron of the Croydon Infirmary on the subject of the nursing staff. Until this week the question in dispute has been discussed in private, but on Tuesday the matter came before the meeting of the Board. It appears that the matron, Miss Julian, declined to sign the certificates of three probationers when their period of service was finished. The Committee responsible for the arrangement then determined to recommend (1) that the immediate and preliminary training of the probationer nurses be entrusted to the ward Sisters; (2) that the medical superintendent be requested to undertake the training of the probationer nurses and the periodical lectures to the charge nurses and probationers. These recommendations were agreed to.

In the meantime, however, the matron had written to the Local Government Board. In her letter she said: "I cannot believe that the Croydon Board of Guardians have the right to depose me from my recognised position by the Local Government Board as superintendent of nurses in the infirmary or to determine that I shall sign no more certificates of training, and that my duties shall be of a purely domestic character. The resolutions passed by the Guardians on September 18th render the Croydon Infirmary no longer a training school for nurses, and this is a monstrous injustice to the present staff of nurses now in training, and also to those who have left us, and who, on the strength of the certificate granted by the authorities, are practising their profession throughout the country. No reason whatever has been assigned by the Guardians for this drastic treatment. Presumably, therefore, it is for 'conscience sake,' as I refused to sign the certificate of three women who were, in my opinion, utterly unworthy of the name of nurses, and whose general conduct was eminently unsatisfactory. . . . I have given ten years of my life to this institution, and I cannot bear to think of it being dragged from its high position in the nursing world by an open scandal. I therefore pray that your honourable Board will give this matter your serious consideration to avert this calamity."

In reply to a member, the CHAIRMAN said, the matron had always hitherto signed these certificates. She refused to sign three on the grounds that the probationers were not competent, but the medical superintendent thought otherwise.

Mr. SHIRLEY (Chairman of the Infirmary Committee) said the matron had not complained of these probationers during their term of three years.

It was decided to report the whole of the circumstances to the Local Government Board.

### Appointments.

KIDDERMINSTER ISOLATION HOSPITAL.—Miss L. A. Upton has been appointed nurse matron. She was trained at Monsall Fever Hospital, Manchester, and the Royal Infirmary, Manchester, and has since been sister at the Hull Sanatorium and Plaistow Hospital; assistant matron, Hornsey Isolation Hospital; sister, Borough Hospital, Wolverhampton; and matron, Kingsthorpe Isolation Hospital.

WESTERN HOSPITAL, TORQUAY.—Miss Nora F. Watts has been appointed matron. She was trained at Westminster Hospital, where she was subsequently sister. She has since been sister female wards of the Princess Alice Hospital, Eastbourne; sister medical and surgical wards, Royal Devon and Exeter Hospital, Exeter, and from 1898 night superintendent. Miss Watts has performed the duties of both matron and assistant matron at the Devon and Exeter Hospital.

### Everybody's Opinion.

[Correspondence on all subjects is invited, but we cannot in any way be responsible for the opinions expressed by our correspondents. No communication can be entertained if the name and address of the correspondent is not given, as a guarantee of good faith but not necessarily for publication, or unless one side of the paper only is written on.]

### ALONE IN LONDON.

"DRED" writes: Can you spare a little space for my first experience of the great London? I was obliged to go up to consult a specialist, and for economy, and because I knew no one in London, I wished to return as soon as my business was over. After travelling from 9.30 in the morning to 5 in the afternoon, I went to a so-called hotel for nurses, expecting I could have a meal, rest, and prepare myself for my interview. I saw the manageress, who informed me she could do nothing for me, and recommended me to what she called an A B C. So, sick at heart, hungry, and very nervous, I was turned out into the street, after some explanation about a club, which I was too tired to take in. I found a good friend, however, in the gentleman I went to see, to whom I told my tale, and he and his wife made my short stay very pleasant. Surely there must be places in London where nurses up for a few hours can be accommodated. One thing, however, surprised and pleased me—i.e., the kindness and politeness of the 'busmen.

*** If the managers of the institution referred to do not wish to cater for travellers the use of the word "hotel" in such a case is clearly misleading; but with regard to clubs, a nurse who is not a member cannot expect to be admitted to the privileges which are only enjoyed by members.—ED. *Hospital.*

### "A POOR-HOUSE NURSE ON HER DIGNITY."

"SUPERINTENDENT" writes: With reference to the paragraph in the NURSING MIRROR headed "A Poor-House Nurse on Her Dignity," may I relate my experience before the West Bromwich Guardians? Some four or five years ago I was a candidate for the post of superintendent nurse at the West Bromwich Infirmary, and was selected with four others to appear before the board. When called into the boardroom I was requested to *stand* beside the chairman, who, with the clerk, was seated, so far as I recollect, at a table on a kind of raised platform or daïs. There I stood in full view of the men who were seated below, the target of their critical inspection. I felt so indignant at this treatment that my manner, as I was well aware, was the reverse of conciliatory. I did not therefore expect to be appointed, and retired indeed with the determination that if I were I would refuse the post. Yet in retrospect I can believe that the discourtesy was not intended as such; but my experience on that and some other occasions has taught me that the treatment of candidates by some boards of guardians is far from what it ought to be.

### BETWEEN DEATH AND BURIAL.

"M." writes: Much discussion is going on at present upon the above subject, both in the medical and daily papers, and surely most people will agree with the suggestion that burial should be compulsory within a certain period. Those of us who work amongst the poor see the mania they have for clinging to their dead in spite of all our endeavour to teach them that thus they are endangering the living. My first experience of this unhealthy craze is indelibly impressed upon my memory. I was attending a midwifery case. The home was one of the poorest, and the mother lay in the only bedroom, which was also occupied by the rest of the family. The child died at birth, and before leaving I had prepared the little corpse for its coffin, and thought I had done all that was necessary with regard to it. *Five* days afterwards, upon paying my usual visit, the air of the room seemed to overpower me (at the best of times it was never sweet), and upon looking around, endeavouring to discover the cause, I found the little corpse lying in an old box under the patient's bed. By dint of much threatening I at last made the father promise the burial should take place before evening. Some hours afterwards I met him with the box under his arm. He touched his cap, sullenly saying, "You see I'm keeping my promise, Nurse." This episode (if one may call it so) was a lesson to me, and I trembled when I thought of what it might have meant for that poor mother.

### For Reading to the Sick.

"LET the words of my mouth and the meditation of my heart be always acceptable in Thy sight."—Ps. xix. 14.

THOUGHT alone is Eternal! Time thralls it in vain.
For the Thought that springs upward and yearns to regain
The pure source of spirit—there is no TOO LATE.—*Lytton.*

"Guard well thy thought! Our thoughts are heard in Heaven."—*Young.*

UNLESS Thou show to us Thine own true way,
No man can find it: Father, Thou must lead!
Do Thou, then, breathe these thoughts into my mind
By which such virtue may in me be bred
That in Thy holy footsteps I may tread!
The fetters of my tongue do Thou unbind,
That I may have the power to sing of Thee
And sound Thy praises everlastingly!—*M. Angelo.*

ALL thoughts of ill;—all evil deeds,
That have their roots in thoughts of ill;—
Whatever hinders or impedes
The action of the nobler Will;—
All these must first be trampled down
Beneath our feet, if we would gain
In the bright fields of fair renown
The right of eminent Domain.—*Longfellow.*

### Reading.

REMEMBER every day that He who gives you the morning does not promise the evening, and when He gives you the evening does not promise the next morning.

Spend, then, every moment of the passing hour in a way which is pleasing to God, as if it were your last, and so much the more carefully, seeing that for every moment you must give the strictest account.

Finally, I advise you to consider that day as lost in which (though you may have transacted much business in it) you have neither gained a victory over some sinful inclination or form of self-will, nor thanked the Lord for all His benefits, and above all for the Sorrowful Passion which He endured for you, and for His Fatherly and sweet chastisements, when He has made you worthy to receive from Him the inestimable treasure of some trial.

When (for instance) you feel very melancholy and depressed in spirits, or are tried by heat or cold or any other bodily pain, lift up your heart to the Eternal Will, which has for your good and happiness appointed you this discomfort, and has arranged the time and duration of it. Then, rejoicing in this manifestation of the love of God, and for the opportunity of serving Him in the way which seems to Him best, say in your heart—"Behold in me the fulfilment of the Divine Will, which has from all eternity lovingly ordered that I should now undergo this trial. Blessed, ever blessed, be my most gracious Lord!"

When any good thought arises in your heart, turn at once to God, and acknowledge that it comes from Him, and give thanks to Him for it.

When you read, seem to see beneath the words your Lord, and receive them as if at that moment they were proceeding out of His Divine Lips.

When you look on the Holy Cross, remember that it is the standard of your warfare; and that, if you forsake it, you will give yourself over into the hands of cruel enemies; but if you follow it, you will enter into Heaven, laden with glorious spoils.—*L. Scupoli.*

### To Nurses.

WE invite contributions from any of our readers, and shall be glad to pay for "Notes on News from the Nursing World," or for articles describing nursing experiences, or dealing with any nursing question from an original point of view. The minimum payment for contributions is 5s., but we welcome interesting contributions of a column, or a page, in length. It may be added that notices of entertainments, presentations, and deaths are not paid for, but, of course, we are always glad to receive them. All rejected manuscripts are returned in due course, and all payments for manuscripts used are made as early as possible at the beginning of each quarter.

### Notes and Queries.

**The Editor is always willing to answer in this column, without any fee, all reasonable questions, as soon as possible.**
**But the following rules must be carefully observed:—**
1. **Every communication must be accompanied by the name and address of the writer.**
2. **The question must always bear upon nursing, directly or indirectly.**

**If an answer is required by letter a fee of half-a-crown must be enclosed with the note containing the inquiry.**

*Dr. Abraham.*

(32) Can you oblige me with the address of Dr. Abraham, whose treatment of "Acne" was recently described in THE HOSPITAL?—*F. V.*

Consult the "Medical Directory."

*Imbecile.*

(33) Would you kindly give me the addresses of several homes and asylums where a boy, aged six years, hopelessly imbecile, could be placed either with or without payment?—*Sister H. R.*

The Earlswood Asylum for Idiots and Imbeciles, Redhill. Apply to the Secretary, 36 King William Street, London, E.C. Also to the Medical Superintendent, Darenth Schools, near Dartford, Kent.

*Sanitary Inspector.*

(34) Will you please tell me where I can study for lady sanitary inspector?—*Anxious.*

Apply to the Secretary, the Sanitary Institute, 72 Margaret Street, W.C.

*Charcoal.*

(35) Can you tell me how charcoal, intended for poultices, is prepared? Are antiseptics added to it? 2. I am using castor oil for eye irritation in smallpox, as mentioned in Humphrey's "Nursing Manual" (Ed. 1889). What are its active principles? and is it a remedy generally recommended by medical men?—*M. de la F.*

1. The charcoal used for poultices is ground by the charcoal makers exactly as if it were going to be used for making gunpowder. If any other antiseptics are to be added they will be ordered. 2. The virtue of castor oil is that it is a non-irritating lubricant. It probably has no curative influence beyond that of separating the irritable surfaces. Castor oil is often used by medical men to relieve the irritation produced in the cornea by foreign bodies, or caused by their removal, in which cases it acts in the same way as a non-irritating protective lubricant.

*Hæmorrhoids.*

(36) Is there a cure for hæmorrhoids without an operation? They appeared recently.—*F. M. B.*

Hæmorrhoids are often recovered from under medical treatment; and still more often under such treatment, without being recovered from, they cease to trouble. There are many reasons, however, which may make a patient prefer to have an operation and get rid of his disease, and among such reasons the first and greatest is hæmorrhage. This should never be allowed to go on.

*Massage in Blocked Vein.*

(37) 1. Can you tell me why, in reducing a swelling caused by a blocked vein in the upper arm by massage, the enlargement or bloc seems to go down towards the elbow instead of upwards to be absorbed? 2. Can you tell me of a book to read referring to such a case?—*Vera.*

The reason why the upper part of the swelling is the first to be reduced is that the complete circulation from heart to capillaries and back again to heart is first restored in those parts which are nearest to the heart, and that it is not until the swelling in these parts is reduced that the circulation in the more distant portions can be properly restored. If *Vera* means that the swelling actually moves downwards, that is to say that, while the upper part becomes reduced, portions lower down, which were not swollen before, begin to puff up, she may be sure that she is using too much force in her massage. *Vera* speaks of reducing a swelling "caused by" a blocked vein. For this purpose massage is very useful. She must remember, however, that massage should never be applied with the object of reducing the swelling in the vein itself caused by phlebitis and consequent clotting. Such a proceeding would be attended with great danger. One of the risks of employing massage in cases of general swelling of a limb caused by clotting in a vein is that it is by no means easy, in all cases, to tell in the midst of the general swelling whether the vein itself has sufficiently recovered to make massage permissible. The floating away of a loosening clot, and its impaction in the heart, is a not altogether uncommon cause of sudden death.

*Address.*

(38) I should be much obliged if you could give me the address of Miss Cayky's Nursing Home, Parson's Green, London.—*Enquirer.*

There is no trace of it in the Post Office Directory.

*Superannuation.*

(39) I should be grateful if you could inform me whether a nurse, after having paid into the Superannuation Fund during her term of office in one appointment, could on accepting another appointment in another Poor-law Infirmary contract out. Some of my friends are inclined to believe that once having paid into the fund there is no chance of getting out of it.—*E. L. O.*

A nurse cannot contract out of the Superannuation Act because she changes her infirmary.

### Standard Books of Reference.

"The Nursing Profession: How and Where to Train." 2s. net; post free, 2s. 4d.
"The Nurses' Dictionary of Medical Terms." 2s.
"Burdett's Series of Nursing Text-Books." 1s. each.
"A Handbook for Nurses." (Illustrated.) 5s.
"Nursing: Its Theory and Practice." New Edition. 3s. 6d.
"Helps in Sickness and to Health." Fifteenth Thousand. 5s.
"The Physiological Feeding of Infants." 1s.
"The Physiological Nursery Chart." 1s.; post free, 1s. 3d.

All these are published by THE SCIENTIFIC PRESS, Ltd., and may be obtained through any bookseller or direct from the publishers, 28 and 29 Southampton Street, London, W.C.

